# Association of GDF-15 and Syntax Score in Patient with Acute Myocardial Infarction

**DOI:** 10.1155/2019/9820210

**Published:** 2019-03-05

**Authors:** Shiqiang Liu, Xu Chen, Hua Wang, Bo Ming, Mingle Wu, Yongjie Wang, Tao Liu

**Affiliations:** Department of Cardiothoracic Surgery, Nanchong Central Hospital, The Second Clinical College of North Sichuan Medical College, Nanchong, Sichuan 637900, China

## Abstract

**Aims:**

GDF-15 is considered to be an important biomarker for cardiovascular events, but the differences in serum GDF-15 levels between acute myocardial infarction (AMI) patients and non-AMI patients warrant further investigation.

**Methods:**

A cohort of 409 subjects was enrolled in the current study. The Syntax score was calculated from the baseline coronary angiography results by using online methods. Blood samples were obtained at the start of the study for an assessment of GDF-15 by using ELISA methods.

**Results:**

Patients with AMI had significantly higher levels of serum GDF-15 (Wilcox test, P < 0.001), Syntax scores (Wilcox test, P = 0.006), and left ventricular ejection fractions (LEVF, Wilcox test, P< 0.001). However, no significant differences were present among the other clinical characteristics. The logistical regression analysis indicated that serum GDF-15 levels (P=0.01534) were independent predictors of non-AMI and AMI after adjusting for age, sex, smoking status, and LVEF.

**Conclusions:**

Elevated serum levels of GDF-15 are independently associated with the risk of MI, and GDF-15 may serve as a protective factor for MI in the cardiovascular system.

## 1. Introduction 

Myocardial infarction (MI), also known as a heart attack, occurs when blood flow decreases or is blocked from reaching a part of the heart, which then causes damage to the heart muscle [1]. In 2014, it was reported that the mortality rate due to cardiovascular diseases (CVD) was 295.63 per 100,000 people in rural areas and 261.99 per 100,000 people in urban areas [2]. National statistics have shown that the death rate due to acute myocardial infarction (AMI) sharply rises after the age of 40 years and that the mortality rate of AMI for patients who are over 85 years old is 95x that of the 40- to 45-year age group [3]. Therefore, normal patient populations who are older than 45 years old can be identified as individuals at high risk for MI and ischemic stroke [4]. Although a high incidence of MI occurs worldwide, the diagnosis of special types of MI has still posed a challenge for clinical practice. In the past several decades, cardiac markers (blood tests for heart muscle cell damage) have been mentioned and detected in patients with MI as assisting in the establishment of diagnoses, as well as in predicting future cardiovascular risks [5]. Numerous biomarkers have been used to determine the presence of cardiac muscle damage, such as troponins, creatine kinase-MB (CK-MB), or myoglobin [6]. Consequently, it has become crucial to validate the specific predictive values of these biomarkers in the specific situation of diagnosing MI before they are used as diagnostic or prognostic markers.

Growth differentiation factor 15 (GDF-15), a protein belonging to the transforming growth factor *β* (TGF-*β*) super family, was first identified as macrophage inhibitory cytokine-1, or MIC-1 [7]. The gene for this cytokine, which is located within chromosome 19p13.11, encodes a type of secreted ligand that binds various TGF-*β* receptors, thereby leading to recruitment and activation of SMAD family transcription factors that regulate gene expression [8]. In normal conditions, GDF-15 is expressed at lower levels in different organs and cell lines [9], but increased expression levels are associated with disease states, such as tissue hypoxia, inflammation, acute injury, and oxidative stress [10, 11]. The function of GDF-15 has not been fully illustrated to date; however, in animal models, it appears to have anti-inflammatory, antiapoptotic, or antihypertrophic effects for protecting against cardiac injury [11, 12]. After the occurrence of MI, the levels of GDF-15 increase within hours in human heart tissue [13]. Investigations from Stergios et al. demonstrated that patients with MI and patients with adverse outcomes had higher GDF-15 levels compared with non-MI patients and that increased GDF-15 levels upon admission were associated with a hazard ratio of 2.1 for death or MI (95%CI: 1.67±2.65, P<0.001) [14]. Similarly, research from Alberto indicated that the 2-year outcomes of patients with acute coronary syndrome who developed major adverse cardiovascular events (MACE) had greater GDF-15 concentrations and syntax scores (P <0.001), compared to patients who did not have these conditions. Additionally, there was a positive but moderate correlation between GDF-15 and the syntax score (R=0.45, P<0.0001) [15]. However, whether GDF-15 is a diagnosis marker or a risk biomarker for AMI remains uncertain. In the current investigation, we aimed to test the hypothesis that serum GDF-15 levels can also be used as markers for distinguishing between AMI and non-AMI patients in a Han Chinese population. We also attempted to analyze the correlation between GDF-15 and the Syntax score for a risk prediction of AMI.

## 2. Materials and Methods

### 2.1. Samples

Four hundred nine samples were obtained from the Second Clinical College of North Sichuan Medical College during the time period of 2014 to 2017. All of the patients were assessed by trained cardiologists by using the 2014 ESC Guidelines for the Management of Acute Myocardial Infarction [6]. In addition, basic clinical characteristics (e.g., age, sex, height, body weight, and BMI), as well as blood and biochemical indicators, were recorded for the following analyses.

The AMI diagnostic criteria (according to the 2007 American Heart Association [AHA] unified definition) included the following: (1) patients who presented symptoms of acute chest pain, which is suggestive of an AMI, (2) myocardial necrosis markers (cTn) which were elevated above the reference, normal upper limit score of 99%, and were accompanied by at least one of several factors, including new ischemic changes (such as new ST-T changes or new left bundle branch blocks), electrocardiography (ECG) changes that are suggestive of a new pathogenesis of Q Wave formations, and imaging evidence (which suggests a newly viable myocardial loss or an abnormal wall motion), and (3) non-AMI diagnostic criteria which were present, such as coronary angiographies that showed no obvious stenotic lesions in the coronary arteries. The exclusion criteria were the following: congenital heart disease, a history of percutaneous coronary intervention within 3 months of the start of the study, pregnancy, a history of cardiac surgery, liver or kidney dysfunction, a history of malignancies, an acute pulmonary embolism, cerebral hemorrhage or cerebral infarction, acute infections, and a history of intravenous drug abuse.

This study was conducted with the approval of the ethics committee of the North Sichuan Medical College. The abilities and reasoning of the patients to understand and make rational decisions were included in the investigation. Subjects with traumatic brain injuries, color blindness, hearing impairments, and stuttering were excluded. All of the participants provided written informed consent.

### 2.2. Procedures

Peripheral blood (5 ml) was collected from all of the participants and was stored at –80°C until analysis. Serum GDF-15 (R&D Systems, Minneapolis, USA) levels were measured by using enzyme-linked immunosorbent assays (ELISAs). The concentrations of GDF-15 were higher than the range of detection and were subsequently diluted 10-fold with dilution buffer and reanalyzed in a portion of the samples.

The total Syntax score for each patient was calculated by using online methods (http://www.syntaxscore.com/calculator/start.htm) [16]. All of the evaluations were conducted by two cardiologists who were blinded to the study patient data.

### 2.3. Statistical

The clinical characteristics were presented as means ± SD (standard deviation), and all of the statistical analyses were conducted by using the R platform (http://www.R-project.org/). The categorical comparison tests for the disease traits (non-AMI and AMI) were analyzed by using the *χ*^2^ test or the Wilcox test, as appropriate. The analysis of the correlation between GDF-15 and the Syntax score was performed with the Pearson test. A logistic regression analysis was used to assess the univariate associations between the clinical characteristics and the end point event. A Bonferroni correction was used to adjust the P values in order to reduce the incidence of type I errors due to multiple tests.

## 3. Results

### 3.1. Demographic Characteristics

Four hundred nine enrolled participations were included in the current investigation, and the baseline clinical characteristics are listed in Table 1. The average age of all of the patients was 56.59 ± 11.33 years old, and 229 of the 409 subjects had smoking habits. Most of the patients had high levels of triglycerides (mean ± SD: 1.82 ± 0.79; normal reference value: < 1.7 mmol/L) and glucose (mean ± SD: 6.39 ± 1.97; normal reference value:3.9-6.1*μ*mol/L, under fasting conditions), which may be associated with the occurrence of acute cardiovascular events.

Within all of the recruited patients, 194 people experienced AMI, which included ST-segment and non-ST-segment elevation acute coronary syndrome (NSTEACS), whereas 215 people did not.  Table 2 shows the comparison of the clinical characteristics between the patients with AMI and non-AMI. Patients with AMI had significantly higher levels of serum GDF-15 (Wilcox test, P < 0.001), Syntax scores (Wilcox test, P = 0.006), and left ventricular ejection fractions (LEVF, Wilcox test, P < 0.001). Other clinical, blood, and biochemical characteristics of the patients who were classified according to the presence or absence of AMI showed no significant differences.

### 3.2. Association between Serum GDF-15 Levels and Syntax Scores in Patients with Myocardial Infarction

**Figure 1:**
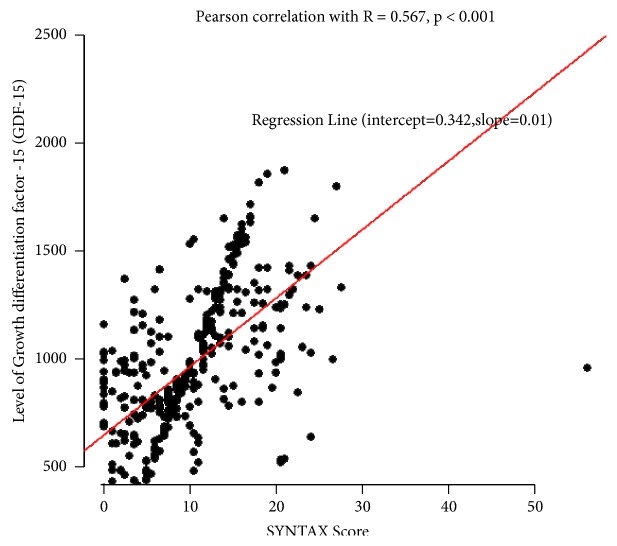
Correlation between GDF-15 levels and Syntax score.

A Pearson correlation was used to examine the association between GDF-15 levels and the Syntax scores in patients with MI. As shown in Figure 1, a moderate correlation existed between serum GDF-15 levels and the Syntax scores (Pearson correlation, R=0.567, P<0.001), and the regression line was depicted with an intercept of 0.342 and a slope of 0.01. From the logistical regression analysis, only serum GDF-15 levels (P =0.01534) were indicated as being independent predictors of non-AMI and AMI (Table 3).

## 4. Discussion

Biomarker testing in clinical practice has been viewed as a supplement to the diagnostic evaluation of patients who suffer from special types of diseases [17]. In the current investigation, it was confirmed that serum GDF-15 levels were correlated with the Syntax scores and that GDF-15 levels were also indicated as being independent predictors of non-AMI and AMI in a Han Chinese population. These results demonstrated that GDF-15 can be used as a confidential biomarker of AMI, rather than solely as a biomarker of cardiac stress.

GDF-15 is expressed at higher levels in placental, prostate, kidney, liver, and colon tissues under physiological conditions; however, in other organs, the expression levels of GDF-15 are low [9]. Similarly, with respect to cell lines, GDF-15 is expressed in endothelial cells, vascular smooth muscle cells, adipocytes, and macrophages. The function of GDF-15 is different in different tissues. For example, GDF-15 that is secreted in fat cells is mainly associated with the inhibition of the differentiation of adipocytes [18]. In activated macrophages, GDF-15 is highly expressed and present in serum that corresponds with various disease conditions [19]. However, under pathological conditions, GDF15 expression can be induced in response to diverse cellular stress signals, such as hypoxia/anoxia, inflammation, acute tissue injuries, and tumor processes. Various conditions, such as pregnancy, tumor, neuronal damage, erythropoiesis, and cardiological status, were companied by increasing GDF-15 levels. When considering the relationship between GDF-15 and cancer, this association has already been discussed to a high degree in other reviews [20]. GDF-15 is also suggested to be involved in the evolution of heart disease and to correspond with acute ischemia, ischemia-reperfusion injury, cardiac hypertrophy, and heart failure.

In 2002, a study by Brown et al. first examined the relationship between GDF-15 levels and cardiovascular events [21]. The results indicated that baseline plasma concentrations of GDF-15 in women were correlated with a higher degree of cardiovascular events (618 versus 538 pg/ml, P =0.0002), even after adjustments for classic cardiovascular risk factors, and that such effects were independent of traditional cardiovascular risk factors and were at least additive to the effects of C-reactive protein. Subsequently, GDF-15 was extensively investigated in relation to the development of heart failure, coronary heart disease, atrial fibrillation, and MI. In ventricular hypertrophy or hypertensive heart disease, it was suggested that the thickness of the posterior wall, serum norepinephrine levels, and the left ventricular mass were positively correlated with elevated levels of GDF-15, which demonstrated the possible cytoprotective effects of GDF-15 [22]. Wiklund et al. demonstrated that elevated serum GDF15 levels were predictors of overall mortality in the multivariate analysis and that this result was independent of age, BMI, and smoking status in a study of 815 Swedish males who were 46 to 80 years old and, in parallel, an independent cohort of 324 twins [23]. The potential mechanism of the relationship between GDF-15 levels and mortality still needs to be validated by more studies. However, the fact that serum GDF-15 levels can predict the progress and prognosis of cardiac events seems to have reached a consensus. Patients with non-ST-segment elevation myocardial infarction benefited from interventional therapy when serum GDF-15 levels were greater than 1200ng/L [24]. During the progress of coronary heart disease, serum GDF-15 levels were closely related to the aggravation of the disease, which suggested a higher incidence of acute cardiac events. More importantly, it has been reported that GDF-15 may be an independent risk factor for AMI. Stergios et al. [14] measured the serum levels of GDF-15 for 1,818 enrolled patients with MI. Patients with MI had higher GDF-15 levels compared with non-MI patients (P <0.001), and the increased GDF-15 levels upon admission were associated with a hazard ratio of 2.1 for death or MI (95%CI: 1.67±2.65, P <0.001) in a model that was adjusted for age and sex. The current investigation first compared the GDF-15 levels between patients with AMI and non-AMI. The results showed that serum levels of GDF-15 in the AMI group were significantly higher than the non-AMI group and that increases in GDF-15 levels can used as an independent predictor for AMI, even after adjustments for age, sex, smoking status, Syntax scores, and LVEF, which further indicated that GDF-15 may function as a monitoring index of myocardial infarction in certain application prospects.

Two limitations must be mentioned with respect to the current investigation. First, the sample size of this study was relatively small, which limited the power to detect the correlation between GDF-15 and the Syntax score. Further studies should include more samples. Second, we did not consider the possible interferences of confounding factors on the relationship between GDF-15 and AMI, which requires further investigation. Additionally, the potential mechanism underlying the association of GDF-15 with AMI has not been thoroughly elucidated to date.

## 5. Conclusions

Our current investigation suggested that elevated serum levels of GDF-15 are independently associated with MI and that GDF-15 may function as a protective factor for MI in the cardiovascular system.

## Figures and Tables

**Table 1:** Baseline level of 409 samples with acute coronary syndrome.

Variables (N=409)	Values (Ratio/Mean ± SD)
Age (years)	56.59 ± 11.33
Sex (Female/male)	229 (55.99%) / 180 (44.01%)
Smoker	221 (54.03%)
Total cholesterol (TC)	4.53 ± 1.02
Triglycerides (TG)	1.82 ± 0.79
High density lipoprotein cholesterol (HDL-C)	1.19 ± 0.74
Low density protein cholesterol (LDL-C)	2.64 ± 0.68
Serum creatinine (CR)	67.82 ± 17.52
Glucose (GLU)	6.39 ± 1.97
Creatine kinase (CK)	665.38 ± 810.64
White blood cell count (WBC)	8.88 ± 3.72
Hemoglobin (Hb)	129.62 ± 16.74
Platelet count (PLT)	236.91 ± 58.43
Left ventricular ejection fraction (LVEF)	50.82 ± 8.64
Growth differentiation factor -15 (GDF-15)	964.32 ± 352.16
SYNTAX Score	11.34 ± 8.67

**Table 2:** Comparison characterization between patients with non-AMI and AMI.

Variables	Non-AMI (n=194)	AMI (n=215)	P value
Age (Years, Mean ± SD)	59.14 ± 11.47	56.52 ± 10.14	0.169
Sex (Female/male)	105/89	124/91	0.534*∗*
Smoking (Smoker/Non-smoker)	96/98	115/100	0.478*∗*
Total cholesterol (TC)	4.85 ± 0.98	4.84 ± 0.86	0.934
Triglycerides (TG)	1.69 ± 0.82	1.70 ± 0.74	0.472
High density lipoprotein cholesterol (HDL-C)	1.17 ± 0.25	1.22 ± 0.89	0.339
Low density protein cholesterol (LDL-C)	2.53 ± 0.83	2.57 ± 0.74	0.236
Serum creatinine (CR)	70.54 ± 21.56	69.56 ± 16.99	0.369
Glucose (GLU)	6.23 ± 1.29	6.35 ± 1.65	0.447
Creatine kinase (CK)	575.63 ± 784.59	769.7 ± 1011.05	0.163
White blood cell count (WBC)	9.04 ± 3.88	8.69 ± 3.58	0.724
Hemoglobin (Hb)	137.67 ± 19.76	140.98 ± 15.42	0.289
Platelet count (PLT)	221.5 ± 50.13	242.99 ± 62.08	0.121
Left ventricular ejection fraction (LVEF)	44.36 ± 8.21	50.89 ± 7.53	*<0.001*
Growth differentiation factor -15 (GDF-15)	798.65 ± 197.05	1121.94 ± 406.14	*<0.001*
Syntax Score	7.61 ± 8.19	13.31 ± 10.12	*0.006*

Wilcox or chi-sq test*∗* according to data type.

**Table 3:** Logistical regression analysis of the myocardial infarction happening in 409 patients.

	Estimate	Std. Error	Z value	Pr(>|z|)
(Intercept)	1.45	0.065	21.99	*<0.001*
SYNTAX	0.0028	0.0045	0.637	0.5248
GDF_15	0.0019	0.0007	1.485	*0.0134*
Age	-0.0147	0.038	-0.386	0.67
Sex	0.37	1.025	0.352	0.73
Smoking	-0.55748	1.098	-0.508	0.512
LVEF	0.232	0.067	0.507	0.056

## Data Availability

The data used to support the findings of this study are available from the corresponding author upon request.
